# Longitudinal long COVID symptoms in Japanese patients after COVID-19 vaccinations

**DOI:** 10.1016/j.jvacx.2023.100381

**Published:** 2023-09-01

**Authors:** Kensuke Nakagawara, Atsuho Morita, Ho Namkoong, Hideki Terai, Shotaro Chubachi, Takanori Asakura, Hiromu Tanaka, Fumimaro Ito, Emiko Matsuyama, Masanori Kaji, Ayaka Saito, Hatsuyo Takaoka, Masahiko Okada, Keeya Sunata, Mayuko Watase, Kazuma Yagi, Keiko Ohgino, Jun Miyata, Hirofumi Kamata, Ichiro Kawada, Keigo Kobayashi, Toshiyuki Hirano, Takashi Inoue, Junko Kagyo, Tetsuya Shiomi, Kengo Otsuka, Naoki Miyao, Toshio Odani, Rie Baba, Daisuke Arai, Ichiro Nakachi, Soichiro Ueda, Yohei Funatsu, Hidefumi Koh, Kota Ishioka, Saeko Takahashi, Morio Nakamura, Toshiro Sato, Naoki Hasegawa, Yuko Kitagawa, Takanori Kanai, Makoto Ishii, Koichi Fukunaga

**Affiliations:** a Division of Pulmonary Medicine, Department of Medicine, Keio University School of Medicine, Tokyo, Japan; b Department of Infectious Diseases, Keio University School of Medicine, Tokyo, Japan; c Department of Clinical Medicine (Laboratory of Bioregulatory Medicine), Kitasato University School of Pharmacy, Tokyo, Japan; d Department of Respiratory Medicine, Kitasato University, Kitasato Institute Hospital, Tokyo, Japan; e Department of Internal Medicine, Sano Kosei General Hospital, Tochigi, Japan; f Department of Internal Medicine, Keiyu Hospital, Kanagawa, Japan; g Department of Internal Medicine, Nihon Koukan Hospital, Kanagawa, Japan; h Department of Rheumatology, National Hospital Organization Hokkaido Medical Center, Hokkaido, Japan; i Pulmonary Division, Department Internal Medicine, Saiseikai Utsunomiya Hospital, Tochigi, Japan; j Department of Internal Medicine, Saitama Medical Center, Saitama, Japan; k Division of Pulmonary Medicine, Department of Internal Medicine, Tachikawa Hospital, Tokyo, Japan; l Department of Pulmonary Medicine, Tokyo Saiseikai Central Hospital, Tokyo, Japan; m Department of Organoid Medicine, Keio University School of Medicine, Tokyo, Japan; n Department of Surgery, Keio University School of Medicine, Tokyo, Japan; o Division of Gastroenterology and Hepatology Department of Internal Medicine, School of Medicine, Keio University, Tokyo, Japan; p Department of Respiratory Medicine, Nagoya University Graduate School of Medicine, Nagoya, Japan

**Keywords:** SARS-CoV-2 infection, Vaccination, post-COVID-19 syndrome

## Abstract

We conducted a subgroup analysis of a study on the long-term effects of COVID-19 (long COVID) in Japan to assess the effect of vaccination on long COVID symptoms. We assessed the clinical course of 111 patients with long COVID at the time of vaccination. The follow-up period was one year from the onset of COVID-19 or until the administration of the third vaccine dose. Of the 111 patients, 15 (13.5%) reported improvement, four (3.6%) reported deterioration, and 92 (82.9%) reported no change in their long COVID symptoms after vaccination. The most common long COVID symptoms before vaccination were alopecia, dyspnea, muscle weakness, fatigue, and headache among participants whose symptoms improved. Reduced dyspnea and alopecia were the most frequently reported improvements in symptoms after vaccination. Some symptoms persisted, including sleep disturbance, myalgia, and hypersensitivity. Vaccination did not appear to have a clinically important effect on patients with long COVID symptoms.

## Highlights

• Some Japanese patients with long COVID had improved symptoms after vaccination.
• Symptoms that improved varied and included dyspnea, alopecia, and fatigue.
• Overall, vaccination had no clinically significant effect on long COVID symptoms.

## 1 Introduction

Persistent symptoms of coronavirus disease (COVID-19) have attracted global attention and become a social problem. In a systematic review of approximately 250,000 patients from 57 studies, more than half of the patients reported long-term COVID symptoms (long COVID) [1]. A few studies have found that COVID-19 vaccination can alleviate long COVID symptoms and prevent their onset [2], [3]. In an online survey of long COVID in the United States, approximately 40% of respondents reported that some or all their symptoms resolved after vaccination, and 14% reported worsening of their symptoms [4]. In a similar study of 900 patients with long COVID in the United Kingdom, more than half of the respondents reported an improvement, and approximately 3% reported worsening of their symptoms after vaccination [3]. Hesitation regarding COVID-19 vaccination is common among individuals with long COVID symptoms [5]; thus, more evidence is required. To our knowledge, the relationship between vaccination and long COVID symptoms in Japan has not been reported previously. Therefore, we evaluated the clinical course of long COVID symptoms in Japanese patients after vaccination.

## 2 Methods

**Fig. 1:**
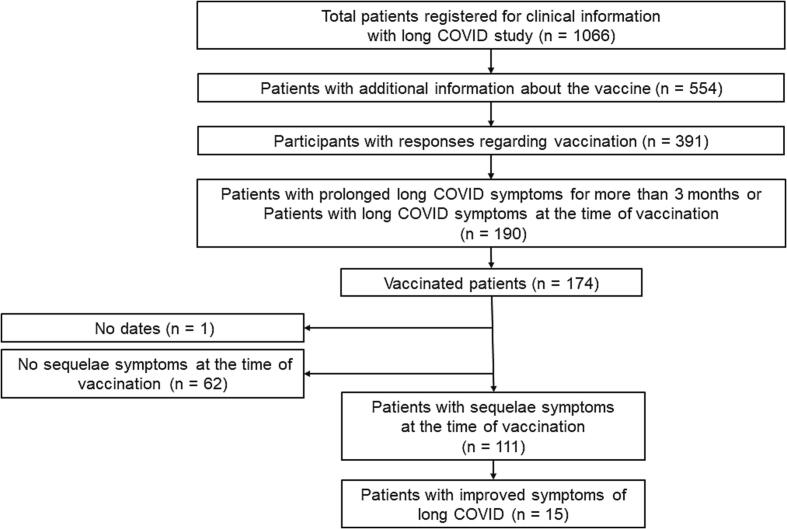
**Study flow chart of patient identification and participant selection** Of the 554 patients who participated in comprehensive surveys of long COVID in Japan, 391 responded to additional questions on COVID-19 vaccination, of whom 174 were vaccinated and 111 had few long-term COVID symptoms at the time of vaccination. Of the 111 participants with long COVID symptoms at the time of vaccination, 15 reported improvements in their symptoms after vaccination.

We conducted a comprehensive nationwide survey of 1,066 patients with long COVID at 27 hospitals throughout Japan from January 2020 to February 2021 [6], with additional questions on COVID-19 vaccination completed by 554 participants. We evaluated the long COVID symptoms in 391 consecutive participants after vaccination. The respondents were asked to select one of three options in the questionnaire regarding their symptoms: recovery, aggravation, or no change. Of the participants who answered the questionnaire on the effect of vaccination on their long COVID symptoms, 190 (48.6%) reported long COVID symptoms for more than three months, and 174 (91.6%) had been vaccinated against COVID-19. After excluding 62 participants without long COVID symptoms at the time of vaccination and one participant with missing information on vaccination dates, 111 adult participants with long COVID symptoms at the time of vaccination were included in this analysis (Fig. 1). The patient backgrounds of the groups whose symptoms were improved or not after vaccination were evaluated by chi-square and t-tests. Statistical significance was set at p less than 0.05. All data were analyzed using the JMP 16 program (SAS Institute Japan Ltd., Tokyo, Japan).

## 3 Results

**Fig. 2:**
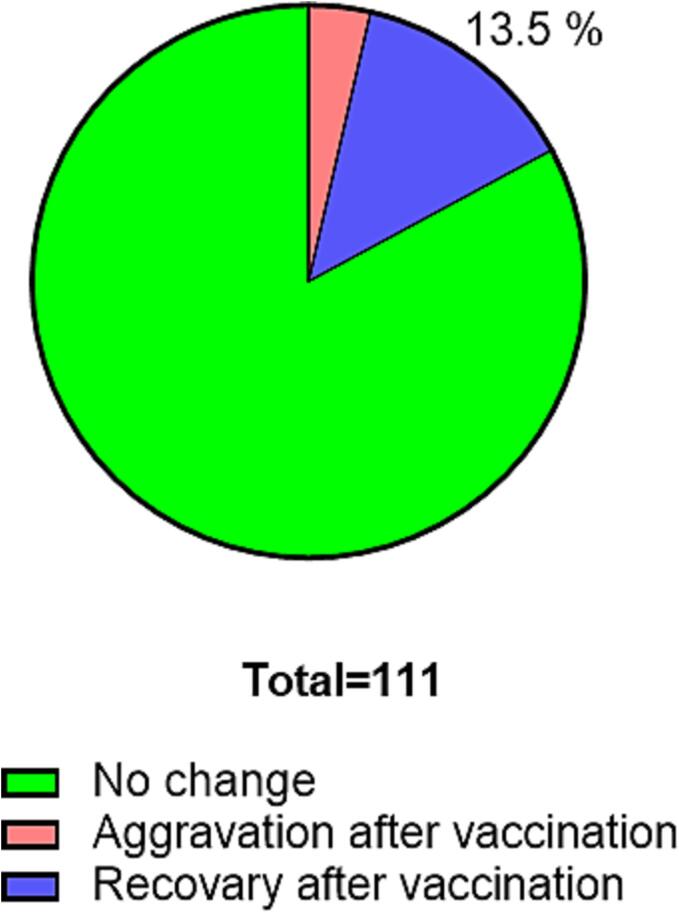
**Details of post-vaccination long COVID symptoms.** Most participants (92/111, 82.9%) reported no change in their long COVID symptoms after COVID-19 vaccination, but 15/111 (13.5%) reported improvement, and 4/111 (3.6%) reported worsening of their symptoms after vaccination.

**Fig. 3:**
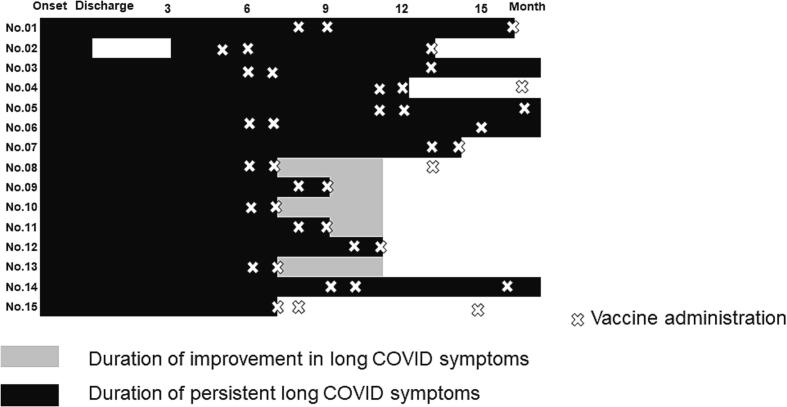

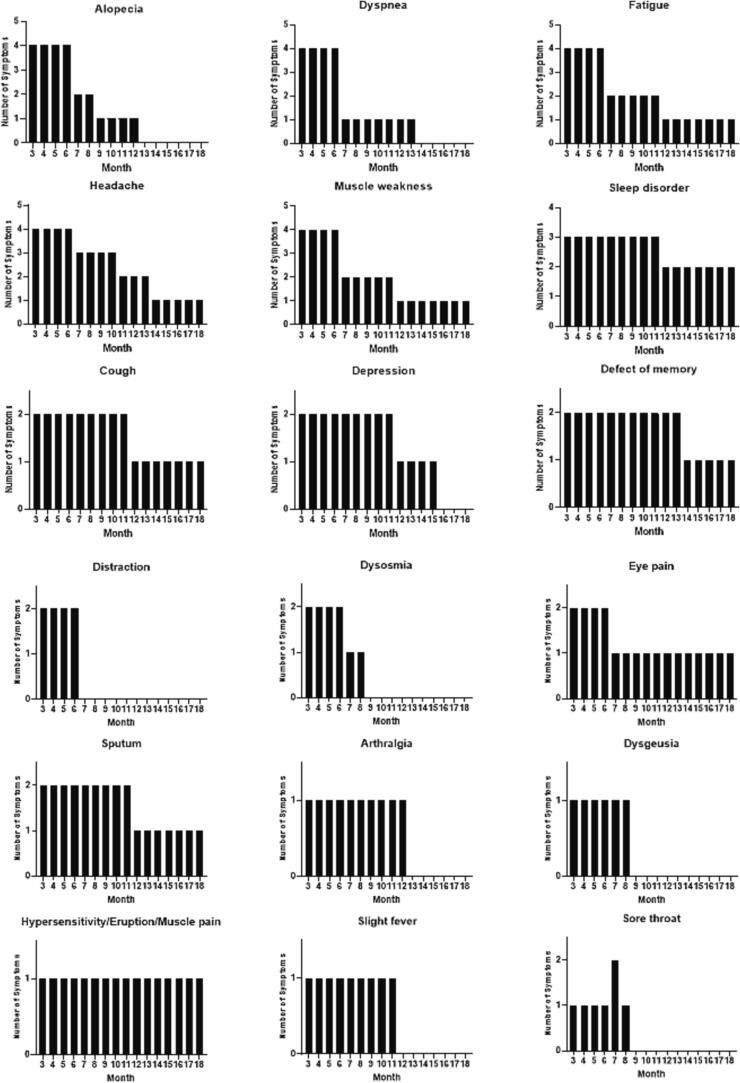
**Clinical course of long COVID symptoms after vaccination (a) Time course of long COVID in vaccinated patients.** Fifteen participants reported resolution of some COVID-19 symptoms after vaccination. Eleven reported complete resolution of all symptoms: one after the first vaccination, eight after the second vaccination, and two after the third vaccination. **(b) Detailed trends in the improvement of long COVID symptoms during clinical course** Participants reported improvements in several types of long-term COVID symptoms after vaccination; dyspnea and alopecia improved most frequently. Symptoms, such as sleep disorders, myalgia, hypersensitivity, eruption, and sputum, persisted until the end of the follow-up period.

Of the 111 participants, 15 reported symptom resolution after vaccination (Fig. 1). The mean number of days (±standard deviation) from COVID-19 onset to the first vaccine dose administration was 298.2 (±114.0) days. The Pfizer and Moderna mRNA COVID-19 vaccines were used in this study. During the first vaccination, 111 patients had long COVID symptoms (94 received the mRNA COVID-19 vaccine BNT162b2 from Pfizer and BioNTech, 16 received mRNA-1273 from Moderna, and 1 patient received an unknown vaccine). During the second vaccination, 107 patients received the vaccine (86 received BNT162b2 from Pfizer and BioNTech, 13 received mRNA-1273 from Moderna, and eight patients received an unknown vaccine). Additionally, 54 patients received a third vaccination (35 received BNT162b2 from Pfizer and BioNTech, 18 received mRNA-1273 from Moderna, and 1 patient received an unknown vaccine). After vaccination, 15 patients reported that their long COVID symptoms had improved, and 4 participants reported worsening of symptoms (Fig. 2, Fig. 3A). The proportion of participants with long COVID symptoms tended to decrease with repeated vaccinations. Eleven participants reported complete resolution of their symptoms after vaccination: one after the first vaccination dose, eight after the second vaccination, and two after the third vaccination (Fig. 3A). In patients whose symptoms improved after vaccination, the most common persisting symptoms were alopecia, dyspnea, muscle weakness, fatigue, and headache. Participants reported improvement in various symptoms after vaccination, of which alopecia and dyspnea were the most frequently reported symptoms that improved (Fig. 3B). Some symptoms including fatigue, dyspnea, and myalgia persisted. There were no statistically significant differences in the clinical characteristics of the participants who did and did not report an improvement in long COVID symptoms after vaccination (Table 1).

**Table 1:** Comparison of clinical backgrounds of patients whose sequelae were ameliorated by vaccine and those were not.

	**Population with no recovery of sequelae after vaccination** **(N = 96)**	**Population with recovery of sequelae after vaccination** **(N = 15)**	***P* value**
Age	55.4 ± 15.5	60.1 ± 14.1	0.27
Sex(male), %	63.5	60	0.79
BMI	24.6 ± 4.8	23.6 ± 4.6	0.49
Smoker, %	14.6	6.7	0.40
Hypertension, %	33.3	40.0	0.61
Diabetes, %	22.9	13.3	0.40
Cardiovascular disease, %	3.1	6.7	0.49
Cancer, %	5.3	0	0.36
Asthma, %	5.3	6.7	0.83
COPD, %	8.4	0	0.24
Hyperuricemia, %	13.7	13.3	0.97
Chronic Liver disease, %	2.1	0	0.57
Chronic Kidney disease, %	4.3	0	0.42
Severe COVID-19, %	14.6	13.3	0.90

## 4 Discussion

To our knowledge, this is the first report on the clinical course of long COVID symptoms after vaccination in Japan. The proportion of participants who reported an improvement in their long COVID symptoms after vaccination was lower than that reported in previous studies conducted in other countries [3], [4]. As in previous studies, participants reported improvement in various symptoms after vaccination [3], [7], and no change in fatigue and dyspnea post-vaccination [3].

A strength of this study is that we were able to accurately determine the precise timing of symptom onset with respect to vaccination during long-term follow-up of patients with long COVID. Moreover, we evaluated the course of the long COVID symptoms until the third vaccine administration. Some previous studies have only evaluated symptoms after one or two vaccinations [7], [8]. However, this study has some limitations. First, the sample size was small, leading to limited statistical power in detecting any potential association between long COVID symptoms and vaccination. Consequently, determining the exact impact of vaccination on long COVID is challenging based solely on these results. Second, the period between symptom onset and vaccination was not uniform across participants. Additionally, the participants in our study may not be representative of the entire population of patients with long COVID. Furthermore, many long COVID symptoms improved over time, making it difficult to accurately isolate the specific effects of vaccination on symptom amelioration. Finally, given the absence of non-vaccinated control group among patients with long COVID, we were unable to establish a clear causal relationship between vaccination and symptom amelioration. However, consistent with the findings of previous studies [3], [8], very few participants reported worsening of long COVID symptoms after vaccination. To address these limitations and gain more robust insights, future research should aim for larger sample sizes, inclusion of control groups, and standardization of the time course to investigate the impact of vaccination on long COVID.

## 5 Conclusion

Most patients experienced no change in long COVID symptoms after vaccination, but approximately 15% reported improvement and less than 5% reported worsening of their symptoms after vaccination.

## Data availability statement

The data that support the findings of this study are available from the corresponding author upon reasonable request.

## Declaration of Competing Interest

The authors declare that they have no known competing financial interests or personal relationships that could have appeared to influence the work reported in this paper.

## Data Availability

Data will be made available on request.
